# Assessment of the consequences of hypothetical nuclear accidents in an advanced PWR, Egypt

**DOI:** 10.1038/s41598-025-92917-6

**Published:** 2025-03-25

**Authors:** M. Abobakr Mohamed, Moamen G. El-Samrah, Mohamed Y. M. Mohsen, A. F. Tawfic, Ahmed Omar

**Affiliations:** Nuclear Engineering Department, Military Technical College, Cairo, Egypt

**Keywords:** Radiation doses, Atmospheric pollutants, TEDE, CEDE, Radioactive releases, Safeguarding, Radiological risks, Environmental impact, Environmental sciences, Risk factors

## Abstract

The pressurised water reactor (PWR) situated on the northern coast of Egypt is one of the world’s most advanced nuclear power plants. This study aims to accurately assess the consequences of potential radioactive releases during two hypothetical nuclear accidents, thereby safeguarding individuals and the environment. The Health Physics Simulation Software HotSpot 3.1.2 was employed to model this reactor’s two hypothetical accident scenarios (AS1 and AS2). The study introduced and explored the immediate and delayed protective measures related to the effective doses at the pertinent distances concerning AS2. It has been found that increasing the effective release altitude was observed to negatively affect both TEDE and ground deposition, while elevated wind speeds proved to be successful in reducing radioactive harm. Consequently, it is recommended that urgent measures be implemented to safeguard the public from exposure to these pathways in the event of significant radiological incidents, even at considerable distances from the source.

## Introduction

When choosing a location for a nuclear power plant (NPP), it is essential to evaluate the potential impact of radiological releases on the surroundings, including the environment, workers, and nearby communities^1^. While normal NPP operations involve releasing small amounts of radioactivity into the environment, certain accidents could lead to considerable releases. These potential radiological impacts need thorough assessment^1,2^. It is well recognized that one of the primary hazards for the public is the release of radioactive substances; therefore, obtaining a licence for a nuclear reactor site necessitates thorough environmental and radiological safety reviews. As a result, simulating and evaluating atmospheric dispersion and the resulting radiation doses are vital steps in formulating effective emergency plans for any critical nuclear accident scenarios^3^.

Various atmospheric dispersion codes and software have been created to carry out essential environmental and radiological safety evaluations in the event of potential nuclear incidents. One such program is the Hazard Prediction and Assessment Capability Code (HPAC), developed by the Defence Threat Reduction Agency (DTRA). It calculates radioactive emissions, ground contamination, airborne pollutant concentrations, and expected population doses, offering precise forecasts for the movement and spread of Chemical, Biological, Radiological, and Nuclear (CBRN) materials and their impact on the public. These predictions aid in crafting effective emergency response plans for hazardous agent releases^4^.

Another dependable tool is the Radioactive Assessment System for Consequences Analysis for Radiological Emergencies (RASCAL), built by the U.S. Nuclear Regulatory Commission’s (USNRC) emergency operations center, to predict probable doses during radiological emergencies. RASCAL determines radionuclide source terms, dose values, and airborne transport events^5^. Additionally, the Lawrence Livermore National Laboratory (LLNL) developed the HotSpot code to promote the safety of populations near nuclear sites, offering precise predictions of ground contamination and expected radioactive doses for people close to the source of the release^6^.

This code calculates dose distribution percentages for 20 radial distances along the centerline across 16 wind direction sectors, handling up to 50 radionuclides to form a mixed source term. HotSpot restricts the maximum downwind distance to 200 km, assuming constant wind speed and direction, though such conditions are unlikely over long distances. The minimum distance is set at 0.01 km due to the applicability of Briggs’ formulas between 0.1 km and approximately 10 km, extendable to 20 or 30 km. Furthermore, in many studies, HotSpot has estimated effective doses up to 100 km, where the accuracy significantly diminishes beyond this range. Despite this, alternative validation methods are lacking, so these formulas are commonly used up to 100 km^6,7^.

The previously mentioned software tools leverage different models for simulating and analyzing nuclear and radiological accidents, considering specific input parameters. These include the accident details, the consequent nuclide source term, as well as terrain features (such as hills, buildings, and bodies of water) and meteorological conditions (including wind direction and speed, temperature, precipitation rates, and ground shine)^8^. Accurate modelling is essential for forecasting the potential routes and dispersion patterns of radioactive material during such accidents, alongside determining the concentrations of released radionuclides and the resultant doses to the public, assuming that all input parameters accurately replicate the actual conditions and context of the site and the accident. Moreover, correct modelling is pivotal for obtaining precise results, which in turn supports reliable radiological and safety evaluations, allowing for appropriate decisions-such as using iodine tablets, sheltering, and evacuation-to protect workers and the public^9,10^.

Multiple mathematical models were created and incorporated into the aforementioned software applications to investigate the movement patterns of radioactive emissions. The Gaussian Plume Model assumes that pollutants are continuously emitted and that wind speed and direction remain constant both spatially and temporally^11,12^. Additionally, the Gaussian Puff Model treats stack emissions as a series of puffs, each with a Gaussian concentration profile extending in all directions and moving according to the prevailing local wind conditions^13^. Another approach, the Stochastic Lagrange Particle Model, posits that numerous particles are released from each source, each taking a random trajectory around the average wind vector^14^. Pollutant concentrations are then estimated by tallying the particles within each air volume^8^.

This study aims to meticulously examine two proposed hypothetical accident scenarios involving a PWR nuclear reactor (VVER-1200). This type of nuclear reactor is located on the northern coast of Egypt, where Egypt’s first NPP, in El-Dabaa city. The choice of this reactor underscores Egypt’s commitment to sustainable energy development. Therefore, the objective is to accurately assess the consequences of the potential release of radioactive materials by estimating the trajectory of the contaminated radioactive plume and calculating the total effective dose equivalent (TEDE) received up to 80 km from the release point. Moreover, determining the activity of deposited radionuclides, and identifying regions requiring intervention through appropriate safety measures. This would facilitate the evaluation of radioactive risk associated with potential accidents at the proposed nuclear power plant and selected site, and identify necessary protective measures to ensure the safety of personnel and the surrounding public. Finally, this research aligns with IAEA’s GSR Part 7^1^, incorporating concepts like precautionary action zones (PAZ) and urgent protective action planning zones (UPZ).

## Methodology and governing equations

HotSpot implements radiation dosimetry methods that have been sanctioned by the International Commission on Radiological Protection (ICRP). These methods are detailed in Federal Guidance Report No. 11 (FGR-11)^15^. HotSpot dose conversion factors (DCF) employ quality factors, such as radiation weighting factors ($w_R$) and tissue weighting factors ($w_T$), to account for the higher efficacy of certain radiation forms and the variable sensitivity of various tissues to cancer induction. In this study, $FGR_{11}$ was utilized as it provides a more precise result through dose equivalent based on the quality factor that is determined as a function of the linear energy transfer, instead of the radiation weighting factor employed in $FGR_{13}$, which is approximately equivalent to the average quality factor. Hence, two hypothetical scenarios (AS1 and AS2) have been modelled and investigated for a PWR (VVER-1200) reactor situated on Egypt’s north coast. These accident scenarios are thoroughly described in Table 1. Moreover, Eq. (1) characterizes the Gaussian plume model, forming the foundation for the HotSpot program’s predictions of TEDE and surface contamination.

**Table 1:** Description of the two accident scenarios modelled.

Accident scenario code	Description (release scenario)	Causes	Probability of occurrence
AS1	Large break of the steam line inside and outside the containment causes all Noble gases, iodine and cesium that exist in the steam generator loop to release to the environment with tiny radioactivity	Thermal fatigue due to repeating heating and cooling, inadequate inspection and maintenance, and high-velocity steam that can cause erosion	$10^{-2}{-}10^{-4}$ per reactor year
AS2	Failure in the instrumentation or control systems that are responsible for activating the low-pressure ECCS during the LOCA event may prevent the system from initiation properly, Consequently, failure in providing the core with adequate cooling	Seismic events or other external hazards, such as floods or fires	$< 10^{-8}$ per reactor year

1 $$\begin{aligned} C(x,y,z,H)= & \frac{Q}{(2\pi \sigma _y\ \sigma _z\ u)} exp\left[ -\frac{1}{2} \left( \frac{y}{\sigma _y}\right) ^2 \right] \nonumber \\ & \Biggl \{ exp \left[ -\frac{1}{2} \left( \frac{(z-H)}{\sigma _z}\right) ^2 + exp\left[ -\frac{1}{2} \left( \frac{(z+H)}{\sigma _z } \right) ^2 \right] \right] \Biggl \} exp \left[ -\frac{\lambda x}{u}\right] DF(x) \end{aligned}$$

Table 2 outlines the description and units of the required input parameters in the governing equation (eq. (1)) of this Gaussian plume model. This model is trustworthy for providing an initial assessment of the radiological impact following the release of radioactive substances into the atmosphere under a clearly defined accident scenario. The present modelling aims to equip emergency response teams and planning personnel with the necessary insights to conduct an accurate assessment and develop appropriate emergency plans for radiological incidents or accidents pertinent to this reactor type and similar facilities^16^.

**Table 2:** Parameters’ description with their corresponding units utilized in the Gaussian plume model governing equation embedded in the HotSpot program.

Parameter	Description	Units
C	Time-integrated atmospheric concentration	(Ci.s)/m$^3$
Q	Source term	Ci
H	Effective release height	m
$\lambda$	Radioactive decay constant	s$^{-1}$
x	Downwind distance	m
y	Crosswind distance	m
z	Vertical axis distance	m
$\sigma _y$	Standard deviation of the integrated concentration distribution in the crosswind direction	m
$\sigma _z$	Standard deviation of the integrated concentration distribution in the vertical direction	m
u	Average wind speed at the effective release height	m/s
DF(x)	Plume depletion factor	–

Table 3 provides the inventory of radionuclides anticipated to be released into the environment under the two proposed accident scenarios. Radionuclides from these unforeseen releases may impact the human body in various external and internal ways. The total effective dose equivalent (TEDE) constitutes the aggregate of the effective dose equivalent (EDE) from external exposures, including submersion, ground shine, and resuspension, alongside the total committed effective dose equivalent (CEDE) from internal exposures, such as inhalation. TEDE encapsulates the overall radioactive exposure from all pertinent pathways in its most comprehensive form^17,18^.

**Table 3:** Activities based on the amounts of radioactive nuclides released to the environment due to the two proposed ASs.

Nuclide	Half-life	Group	AS1 activity (Bq)	AS2 activity (Bq)
kr-85m	04.48 hr	Noble gas	8.57E+09	1.50E+14
kr-87	76.30 min	Noble gas	1.80E+10	1.79E+14
kr-88	02.84 hr	Noble gas	2.49E+10	3.40E+14
Xe-133	05.24 d	Noble gas	6.34E+10	7.92E+15
Xe-135	09.14 hr	Noble gas	1.65E+10	1.42E+14
Xe-138	14.08 min	Noble gas	5.69E+10	2.30E+14
I-131	08.02 d	Halogens	3.28E+08	3.50E+15
I-132	02.29 hr	Halogens	5.65E+08	3.34E+15
I-133	20.80 hr	Halogens	6.86E+08	2.22E+15
I-134	52.50 min	Halogens	7.36E+08	6.09E+14
I-135	06.57 hr	Halogens	5.83E+08	7.54E+14
cs-134	02.06 y	Alkali metal	8.33E+06	1.50E+15
cs-137	30.07 y	Alkali metal	6.14E+06	5.56E+14

The El-Dabaa area has been identified as the designated site for the planned nuclear reactor, according to this study, which covers roughly 50 km and is bounded by the Mediterranean Sea to the north, the western desert to the south, Alexandria governorate to the east, and Marsa Matruh governorate to the west. The coordinates for the El-Dabaa site are $30^{\circ }~56^\prime ~58^{''}$ N and $28^{\circ }~26^\prime ~41^{''}$ E^19,20^. Compared to other parts of Egypt, El-Dabaa region experiences a climate marked by high relative humidity and moderate temperatures throughout the year. The region experiences two seasons: a dry season from May to October and a wet season from November to April, with the highest rainfall occurring in January.

Notably, this region maintains mild temperatures year-round, with an annual average temperature of 20.25 $^\circ$C. Monthly air temperatures range from a low of 18.8 $^\circ$C in February to a high of 21.7 $^\circ$C in August. Over 30 years, the air temperature in El-Dabaa has risen by approximately 0.1 $^\circ$C^21^. Table 4 provides detailed information on the seasonal surface wind directions and speeds over the last 30 years^22^. For the weather model associated with the site under study, meteorological parameters have been selected including a north-west wind direction with a mean speed of 3 meters per second at 10 meters height and stability class D. The receptor height is set to 1.5 meters, with consideration of different A−F stability classes for comparison with standard (rural) terrain types.

**Table 4:** Seasonal wind direction and speed at El-Dabaa site.

Season	Wind direction	Wind speed m/s
Winter	W $(247.5^\circ{-}292.5^\circ)$	$1.5{-}4.5$
Spring	NW $(292.5^\circ{-}337.5^\circ)$	$1.5{-}4.5$
Summer	NW $(292.5^\circ{-}337.5^\circ)$	$1.5{-}4.5$
Autumn	NW $(292.5^\circ{-}337.5^\circ)$	$1.5{-}4.5$

## Result and discussion

### TEDE and ground deposition with different weather stability classes

**Fig. 1:**
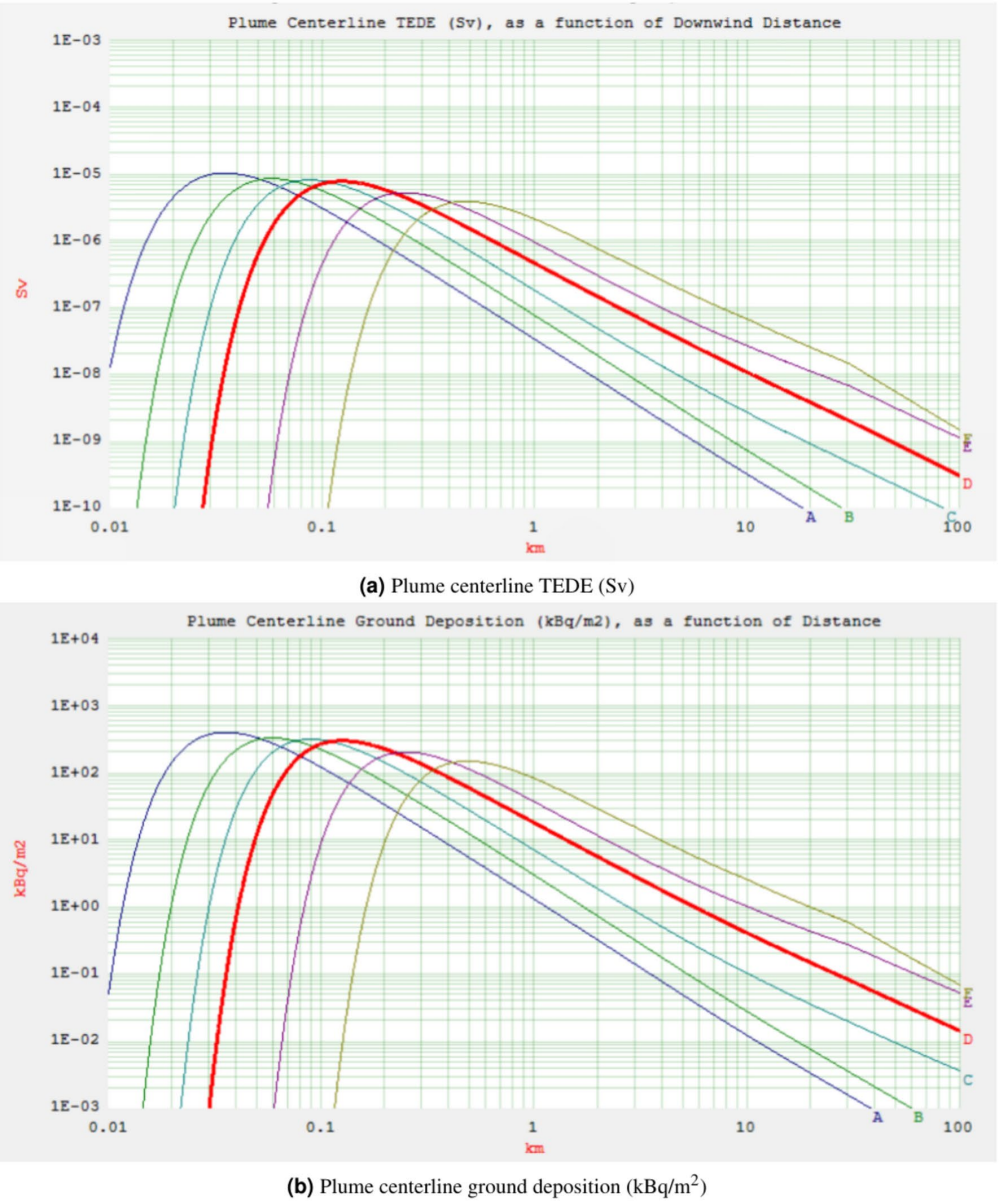
Plume centerline TEDE (Sv) and ground deposition (kBq/m$^2$) as a function of downwind distance (km) for AS1.

**Fig. 2:**
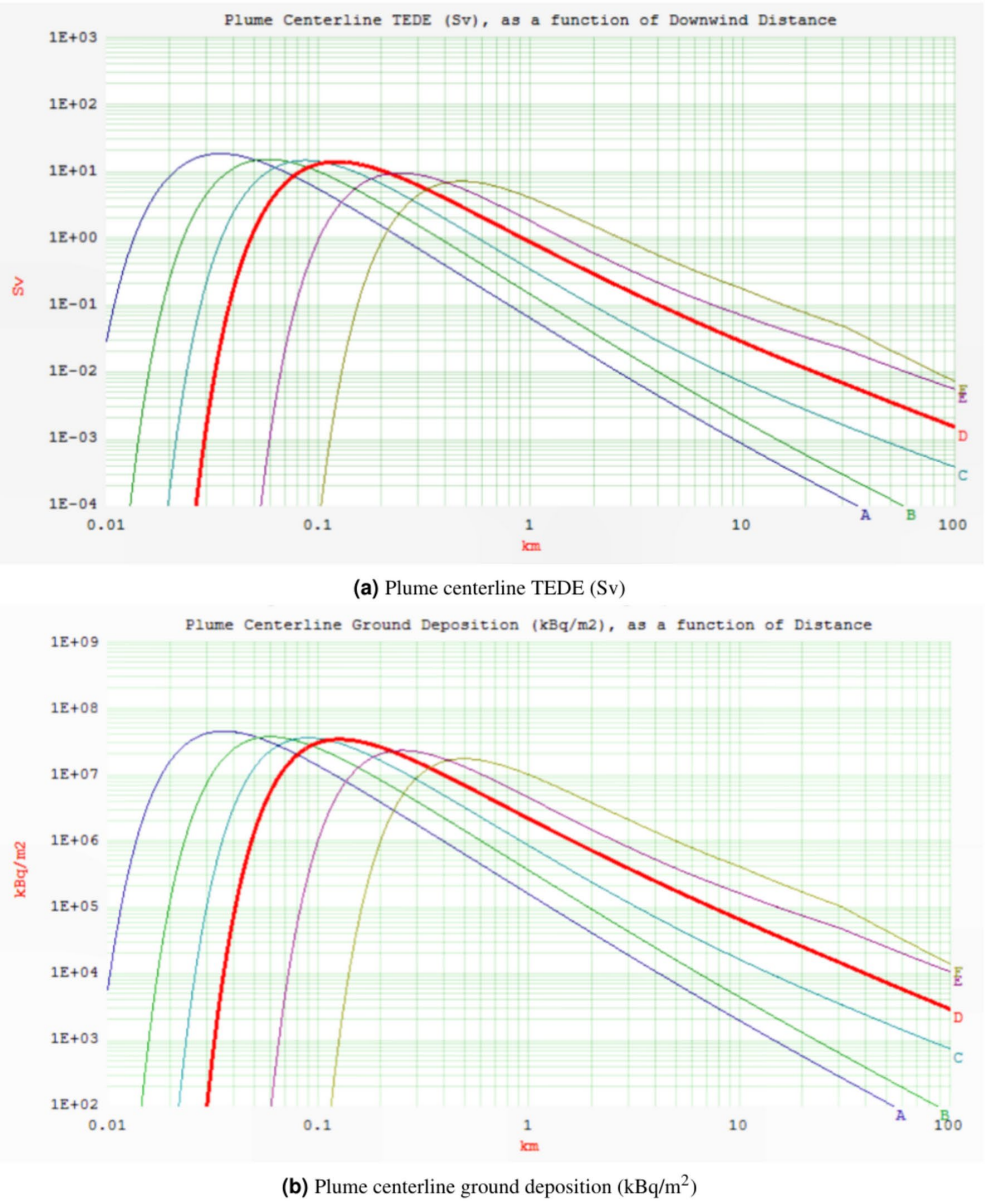
Plume centerline TEDE (Sv) and ground deposition (kBq/m$^2$) as a function of downwind distance (km) for AS2.

The purpose of this part is to provide important takeaways from the two accident scenarios (ASs) that will help with pre-accident preparation and post-accident safety measures. The plume centerline TEDE and ground deposition for both accident scenarios (AS1 and AS2) are shown in Figs. 1 and 2 (the output of the HotSpot programme), respectively. These scenarios consider the downwind distance at an effective release height of 10 m, a northwest wind speed of 3 m/s, and the condition of dry deposition. Starting from A for very unstable weather conditions, moving down the curves are the following: B for moderately unstable, C for somewhat unstable, D for neutral, E for relatively stable, and ultimately, F for moderately stable^23^.

Class A exhibits a TEDE that peaks more rapidly and closer to the source of release compared to other weather stability classes, namely B, C, D, E, and F. The unstable stability classes near the stack yield higher doses due to increased atmospheric turbulence in contrast with the more stable classes. Consequently, while stability classes E and F show minimal doses near the stack, their radiation doses increase as the distance from the stack grows, unlike classes A and B. The mentioned figures (see Figs. 1 and 2) also reveal a reduction in TEDE and ground deposition with an increase in the downwind distance, where weather stability class A demonstrates the lowest TEDE among the examined classes. Enhanced atmospheric stability limits the upward dispersal of pollutants, maintaining lower pollution levels near the stack. Conversely, on sunny, clear days with unstable conditions, pollutants fluctuate vertically with limited mixing. This can lead to sporadic high pollutant concentrations at ground level near the stack. In a neutral atmosphere, represented by stability class D, which is deemed the most likely scenario for the location under investigation-turbulence and diffusion facilitate the horizontal and vertical dispersion of pollutants through the atmospheric layers^24^.

Table 5 presents an overview of the HotSpot simulation results of the two accident scenarios analysed, based on the meteorological conditions relevant to the specified region. Comparative analysis of the modelling results of these two accidents indicates that AS2 results in elevated TEDE (Sv) and ground deposition (kBq/m$^2$). The TEDE (Sv) values of the two accidents are presented alongside their respective arrival timings and distances from the release point. The highest TEDE (Sv) was observed at approximately 0.12 km from the emission source point. In these two scenarios, a worker within the NPP site perimeter would experience a maximum TEDE of approximately 7.83E-06 Sv and 14 Sv due to AS1 and AS2, respectively. Furthermore, permanent public residents located 80 kilometres from the release source will experience a TEDE of approximately 4.4E-10 Sv and 2.0E-03 Sv, respectively.

**Table 5:** The determined TEDE (Sv) of the two accident scenarios at various distances.

Distance [km]	AS1	AS2	Arrival time
0.03	9.60E−10	2.20E−03	< 00:01
0.1	7.20E−06	1.30E+01	< 00:01
0.2	5.70E−06	1.00E+01	00:01
0.3	3.40E−06	6.10E+00	00:01
0.4	2.10E−06	3.90E+00	00:02
0.5	1.50E−06	2.70E+00	00:02
0.6	1.10E−06	2.00E+00	00:03
0.7	8.40E−07	1.60E+00	00:03
0.8	6.70E−07	1.30E+00	00:04
0.9	5.50E−07	1.00E+00	00:05
1	4.60E−07	8.80E−01	00:05
2	1.40E−07	2.90E−01	00:11
4	4.60E−08	1.00E−01	00:22
6	2.40E−08	5.70E−02	00:33
8	1.50E−08	3.80E−02	00:44
10	1.10E−08	2.80E−02	00:55
20	3.80E−09	1.10E−02	01:51
40	1.30E−09	4.70E−03	03:42
60	6.90E−10	2.90E−03	05:33
80	4.40E−10	2.00E−03	07:24

The radioactive release from AS1 poses no significant hazard, even at the release point (the stack), as the calculated Total Effective Dose Equivalent (TEDE) at various distances from the reactor remains substantially below the established safety limits (1 mSv for the public and 20 mSv for workers)^25^. However, the radioactive release associated with AS2 presents a significant hazard in proximity to the release point. The observed TEDE must still be considered, even at a distance of 80 km from the release point. Further analyses regarding AS2 must be conducted and examined, which will be presented in the following sections.

### Various pathways of AS2 radiation exposure

**Fig. 3:**
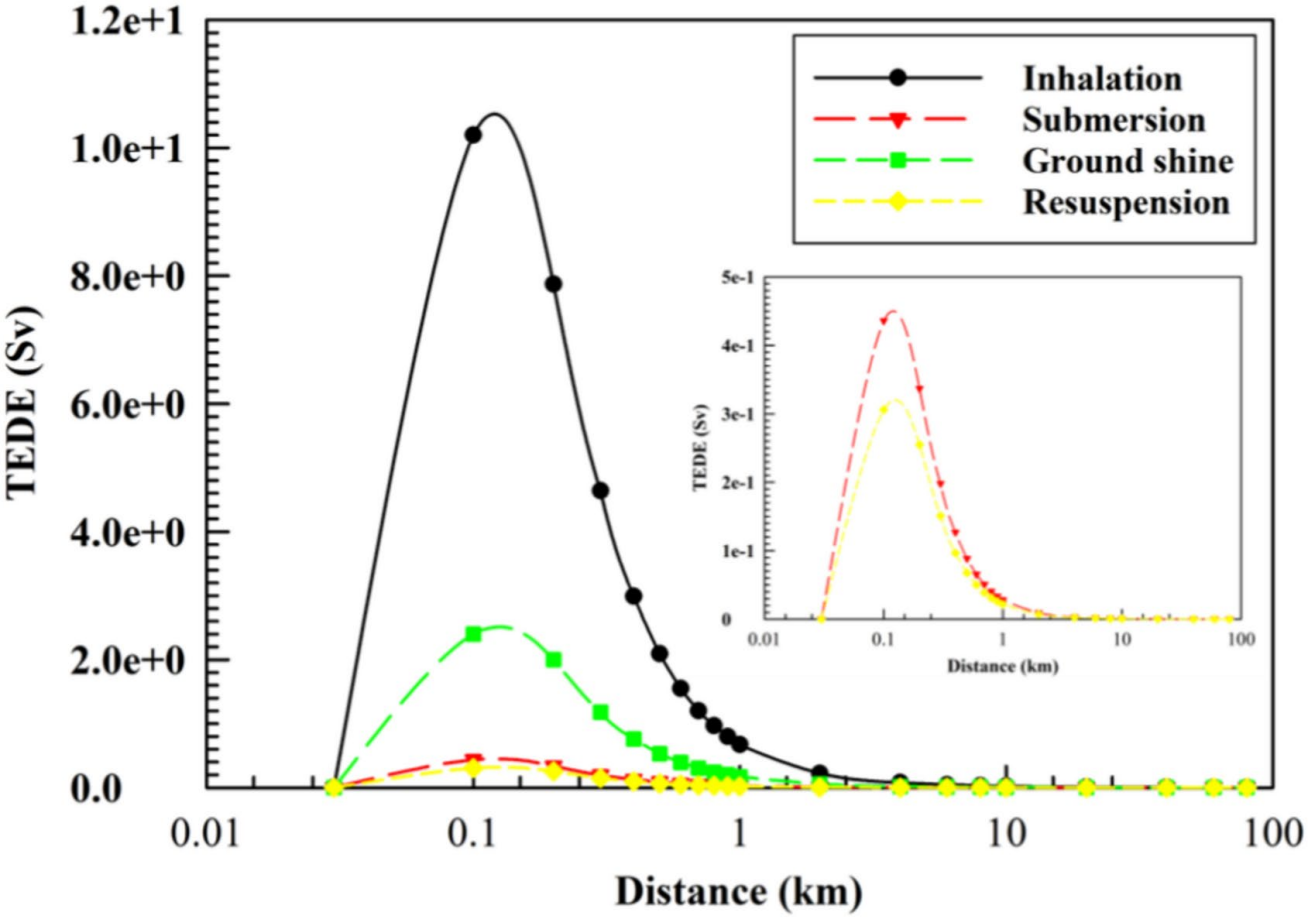
AS2 plume centerline TEDE for different paths as a function of downwind distance.

Various mechanisms of radiological exposure that deliver radioactive doses are identified, including inhalation, submersion, ground shine, and resuspension. The results indicate that inhalation is the predominant mechanism identified by AS2 as presented in Fig. 3. The doses exhibit significant variation based on distance and time, starting at very low levels, increasing rapidly, then sharply declining, followed by a gradual decrease at greater distances. Consequently, it is recommended that urgent measures be implemented to safeguard the public from exposure to these pathways in the event of significant radiological incidents, even at considerable distances from the source.

**Fig. 4:**
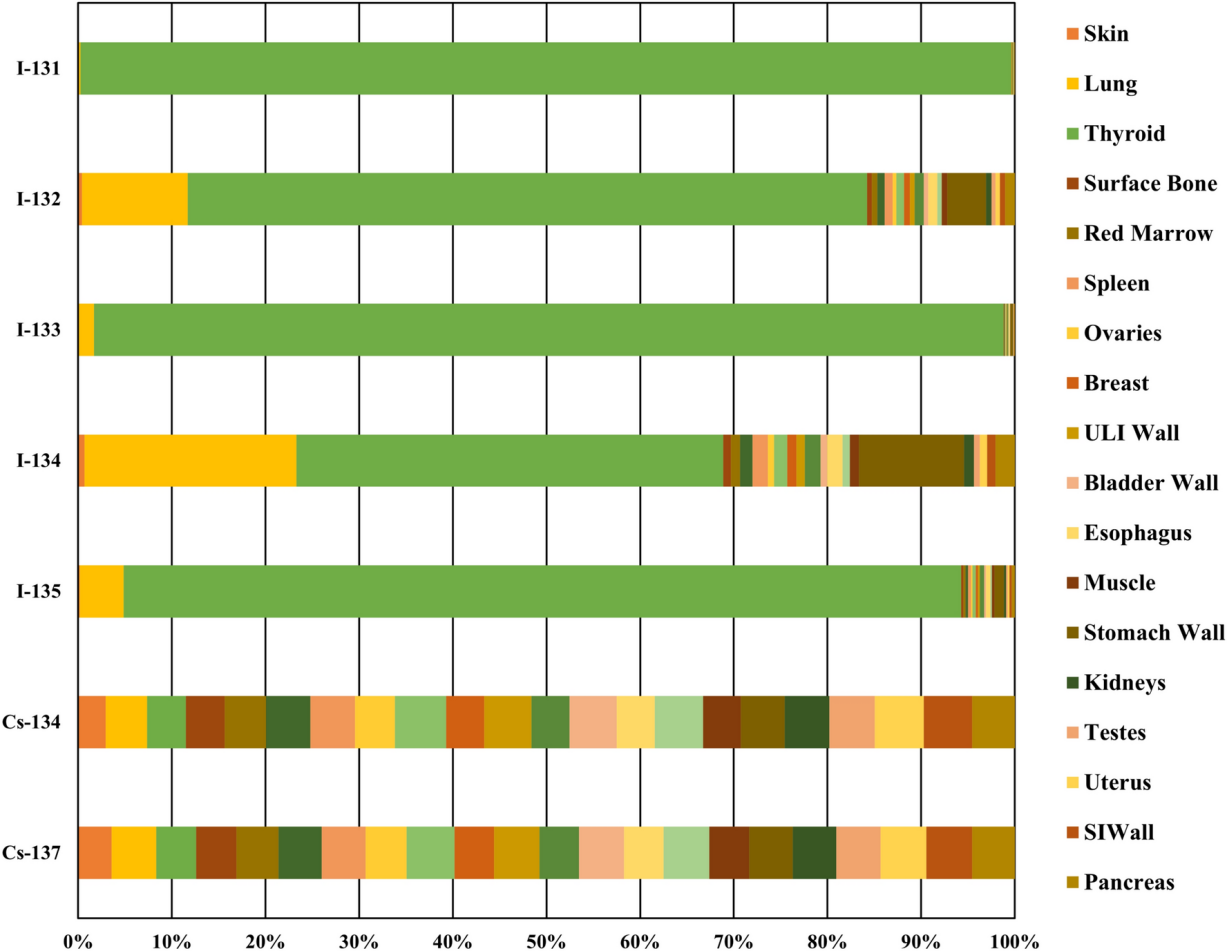
Proportional impact of radionuclides on the CEDE values of organs.

Inhalation is significant; thus, we analysed it using CEDE, which represents the total committed effective dose equivalent that individual tissues or organs will receive over 50 years following material intake via inhalation. The applicable weighting factors, including both tissue and radiation weighting factors, are utilised to calculate a cumulative dose for the whole body. Figure 4 presents radionuclides and their corresponding contributions to the CEDE across the 23 analysed organs. This analysis elucidates the impact of various radionuclides on different tissues and organs. The diagram analysis indicates the substantial impact of radionuclides on various organs. The iodine isotopes (I-131, I-132, I-133, I-134, and I-135) significantly affect the thyroid, as evidenced by the substantial dose accumulation in that region. Moreover, Xenon and krypton, as noble gases, are chemically inert and do not significantly contribute to CEDE due to their lack of distribution within the body. They are rapidly exhaled and do not persist in the body, as CEDE is evaluated over 50 years following the ingestion of radioactive substances.

Radiation exposure poses significant risks to vital organs, with the thyroid gland being one of the most sensitive, particularly to absorbed radiation doses (Gy). This sensitivity has been evidenced in major nuclear accidents such as Chernobyl and Fukushima-Daiichi, where the thyroid was disproportionately affected. The risks associated with radiation exposure to the thyroid are especially pronounced due to its high susceptibility to radioactive iodine uptake, which can lead to severe health complications, including thyroid cancer. This highlights the critical need for focused discussions and studies to evaluate and mitigate the risks of radiation-induced thyroid damage.

Children are particularly vulnerable to thyroid cancer following radiation exposure. Their thyroid glands are still in the developmental stage, making them more prone to absorbing radioactive iodine. Epidemiological studies have documented a marked increase in thyroid cancer cases among children exposed to radiation during nuclear events. The developing nature of their endocrine system, coupled with their higher metabolic activity, accelerates the adverse effects of radiation. Consequently, children represent a high-risk population requiring special attention in radiation safety protocols.

**Fig. 5:**
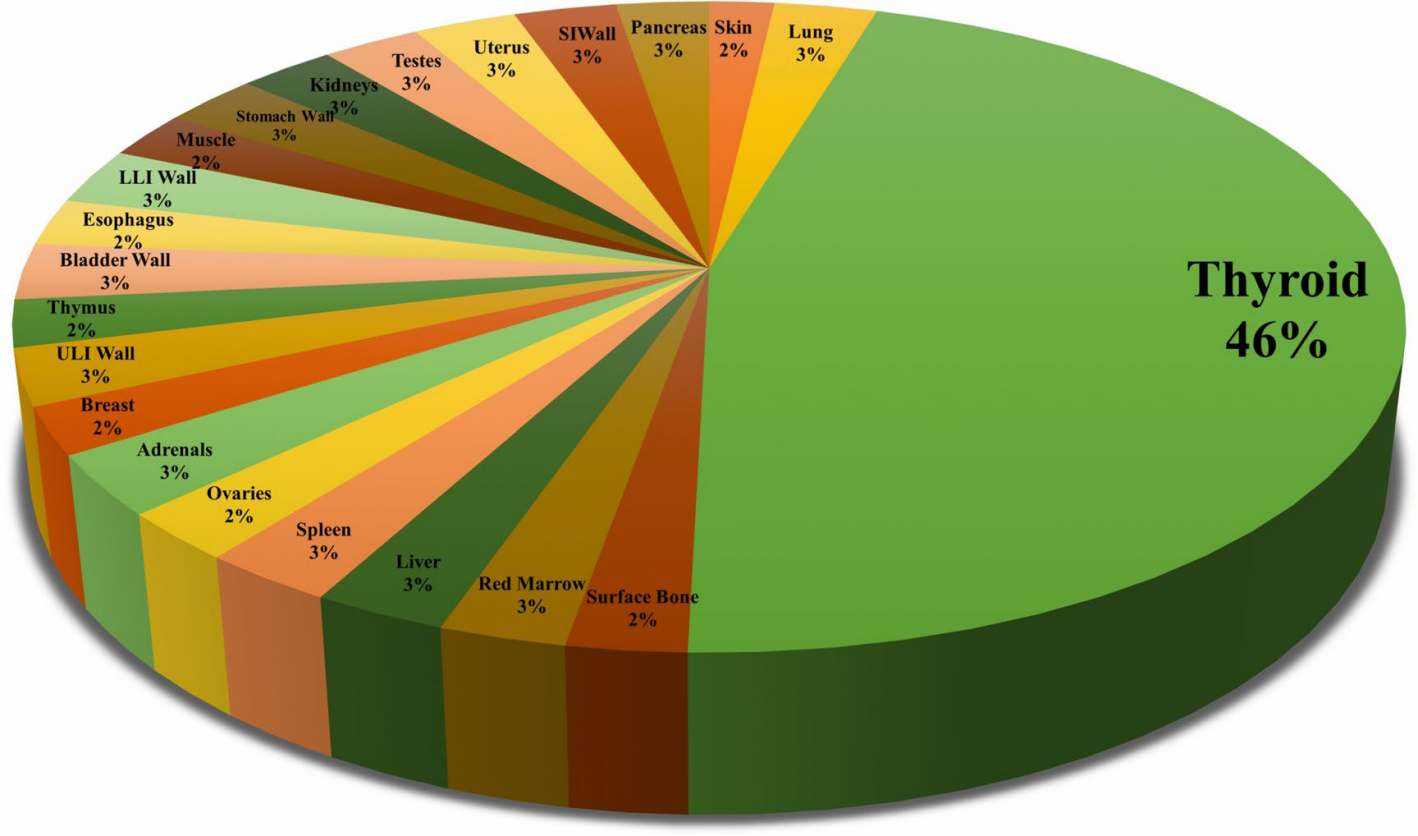
CEDE’s impact on all organs due to radionuclides.

The current study underscores the thyroid’s susceptibility to radiation exposure. The pie chart in Fig. 5 demonstrates that the thyroid accounts for 46% of the total CEDE among affected organs, making it the most impacted organ. Other organs contribute minimally, ranging from 2% to 3% of the total exposure. However, limitations in the data, such as the absence of inhalation dosage information for the brain in the FGR-11 library used in the HotSpot application, may have affected the comprehensiveness of the analysis. Despite these constraints, the findings highlight the disproportionate impact on the thyroid and the urgent need for targeted radiation protection measures.

Based on these findings, several precautionary measures should be implemented to protect infants and children from radiation-induced thyroid cancer. First, emergency iodine supplementation should be made readily available in nuclear accident scenarios to block radioactive iodine absorption. Second, effective evacuation protocols should prioritize shielding children from radioactive exposure. Third, regular monitoring of absorbed doses in children’s thyroids should be conducted during and after exposure events.

### Effect of release height

**Fig. 6:**
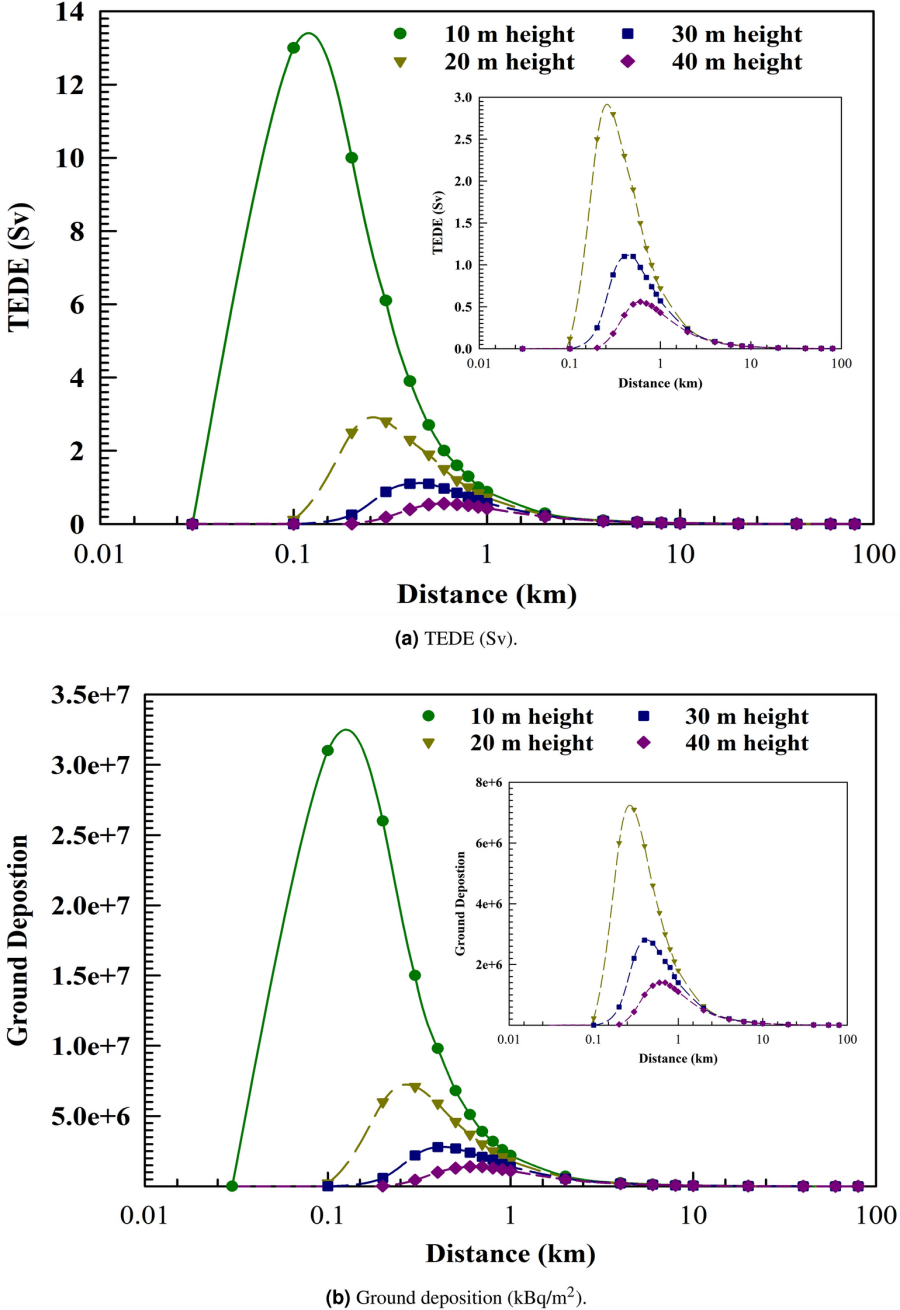
AS2 Plume centerline with different effective release heights.

Figure 6 presents the effective release heights of AS2 at 10, 20, 30, and 40 meters, respectively, under a wind speed of 3 m/s and D stability class. These conditions represent the most probable meteorological scenarios of the selected location on the north coast of Egypt. Increasing the effective release height leads to a reduction in Total Effective Dose Equivalent (TEDE), as higher elevations result in increasing wind speeds, facilitating greater dispersion of air-suspended radionuclides. In the absence of a determinable or calculable effective release height during a nuclear accident, it is typically assumed that the height of the stack of zero is applicable for ground-level releases^17,26^.

### Effect of the wind speed

**Fig. 7:**
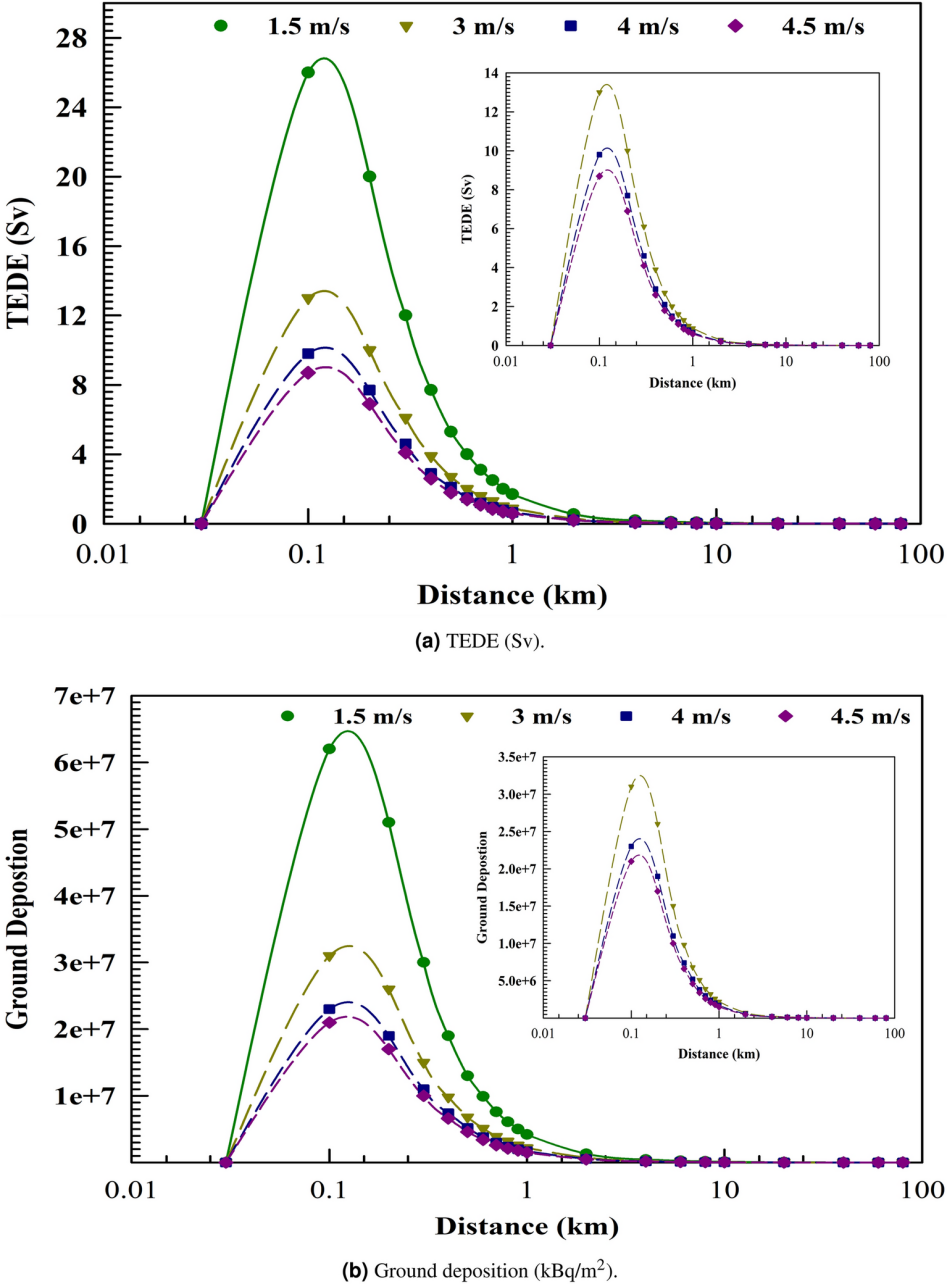
AS2 Plume centerline due to different wind speeds.

Figure 7 demonstrates the effect of increased wind speed, analysing values of 1.5, 3, 4, and 4.5 m/s for AS2, while maintaining a constant release height of 10 m, categorised under the D weather stability class. The findings indicate that, under standard meteorological conditions, TEDE diminishes with an increase in wind speed. The findings suggest that lower wind speeds warrant careful consideration, particularly at distances close to the release source. Low wind speeds reduce air dispersion, thereby increasing the concentration of suspended radionuclides and their probabilities of inhalation and precipitation, ultimately resulting in a significant rise in the TEDE. Higher wind speeds are beneficial for reducing radiological impacts from a nuclear or radiological accident, as they decrease radioactivity deposition and suspension, thereby potentially lowering the doses received.

### Effect of the precipitation (rain)

**Fig. 8:**
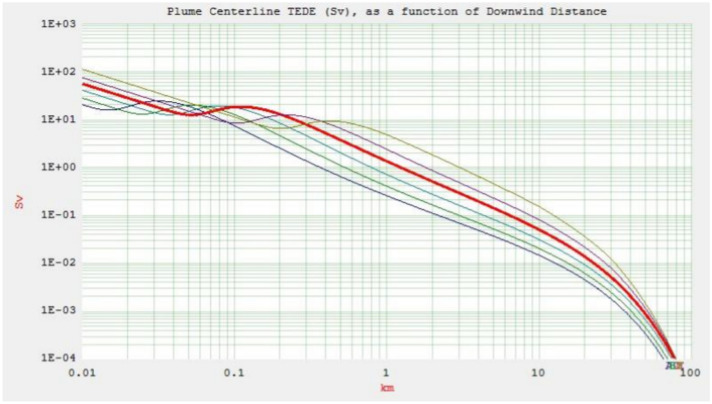
AS2 Plume centerline TEDE (Sv) as a function of downwind distance (km).

**Fig. 9:**
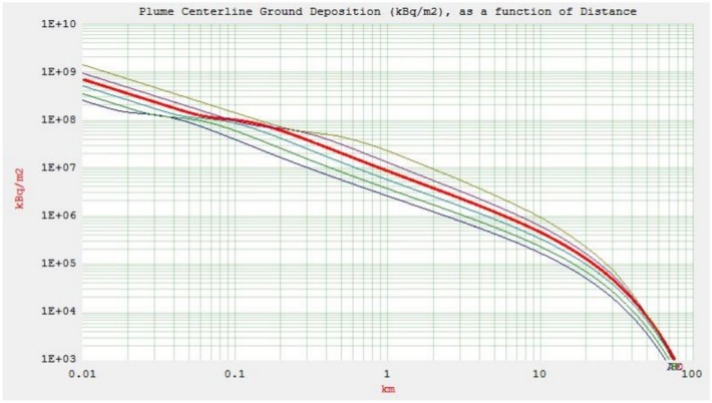
AS2 Plume centerline ground deposition (kBq/m$^2$) as a function of downwind distance.

The impact of rain precipitation on AS2 has been assessed using an assumed rate of 1 mm/hr, which is likely for the region under study during potential rain events with a weather stability class D and a wind speed of 3 m/s. Raindrops can capture gases and particulates from the plume during wet deposition, leading to an increase in ground deposition and, consequently, TEDE, particularly in proximity to the stack, (the source of radioactivity release), as illustrated in Figs. 8 and 9. The impact of precipitation diminishes with greater downwind distance from the radioactivity release point, as the concentration of radionuclides decreases exponentially with increasing distance.

### Influence of the surrounding environment

**Fig. 10:**
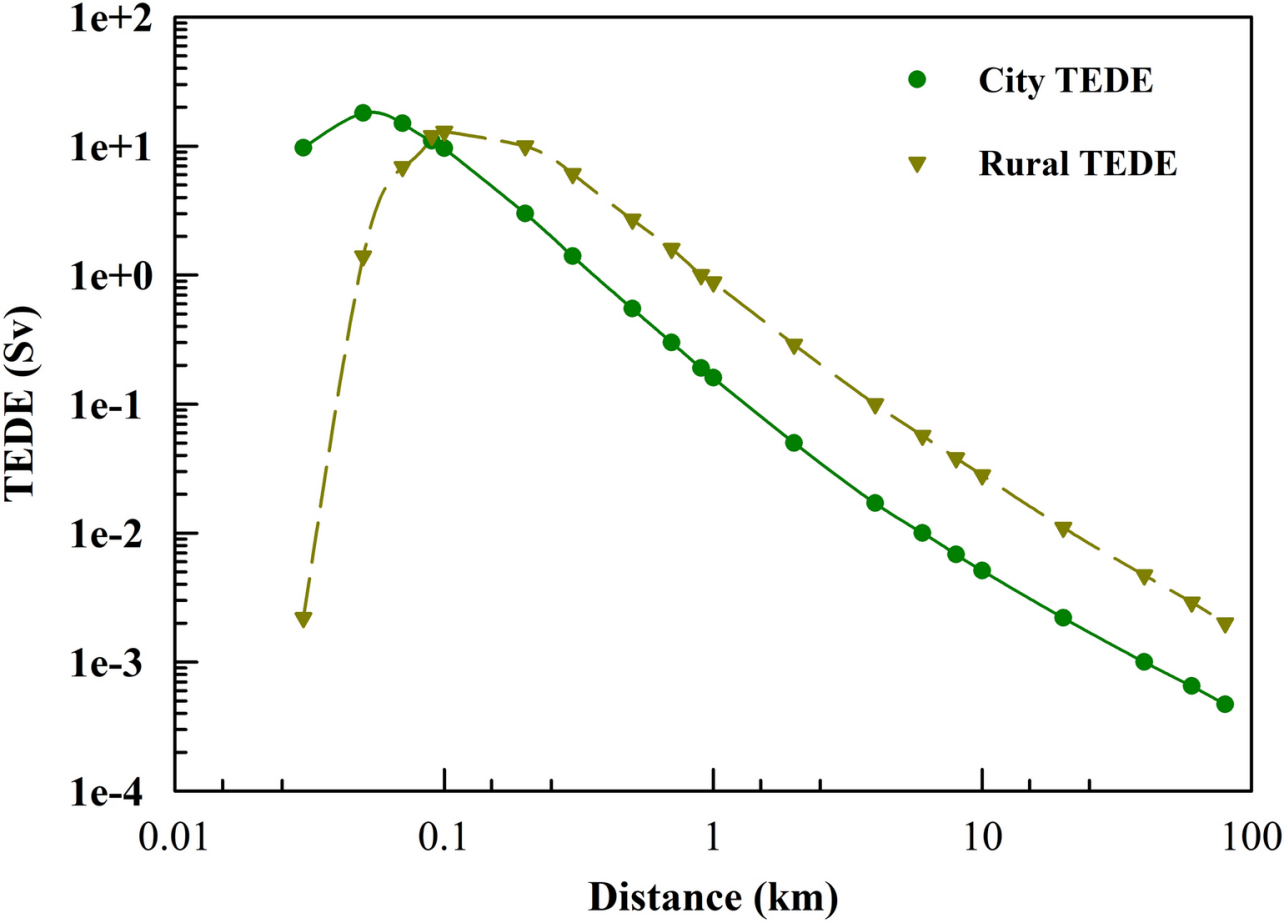
AS2 Plume centerline TEDE (Sv) at different environments.

The simulation was conducted again with the same input parameters, where the surrounding environment has been modified from a plain desert to a metropolitan area to assess its impact on the TEDE. The city terrain factor is anticipated to produce lower concentrations relative to rural or plain desert terrains. In rural and plain desert areas, the maximum Total Effective Dose Equivalent (TEDE) was recorded at 14 Sv at a distance of 0.12 km, while in urban areas, the highest TEDE was estimated to be 18 Sv at a closer distance of approximately 0.05 km, as shown in Fig. 10. This discrepancy arises from the complex wind patterns, barriers, tall buildings, and higher population densities characteristic of urban areas. These factors contribute to a greater reduction in the concentration of deposited radionuclides, while simultaneously causing elevated concentrations near the release point due to the rapid precipitation of these radionuclides.

### Contour plot for designated radiological zones based on AS2

**Fig. 11:**
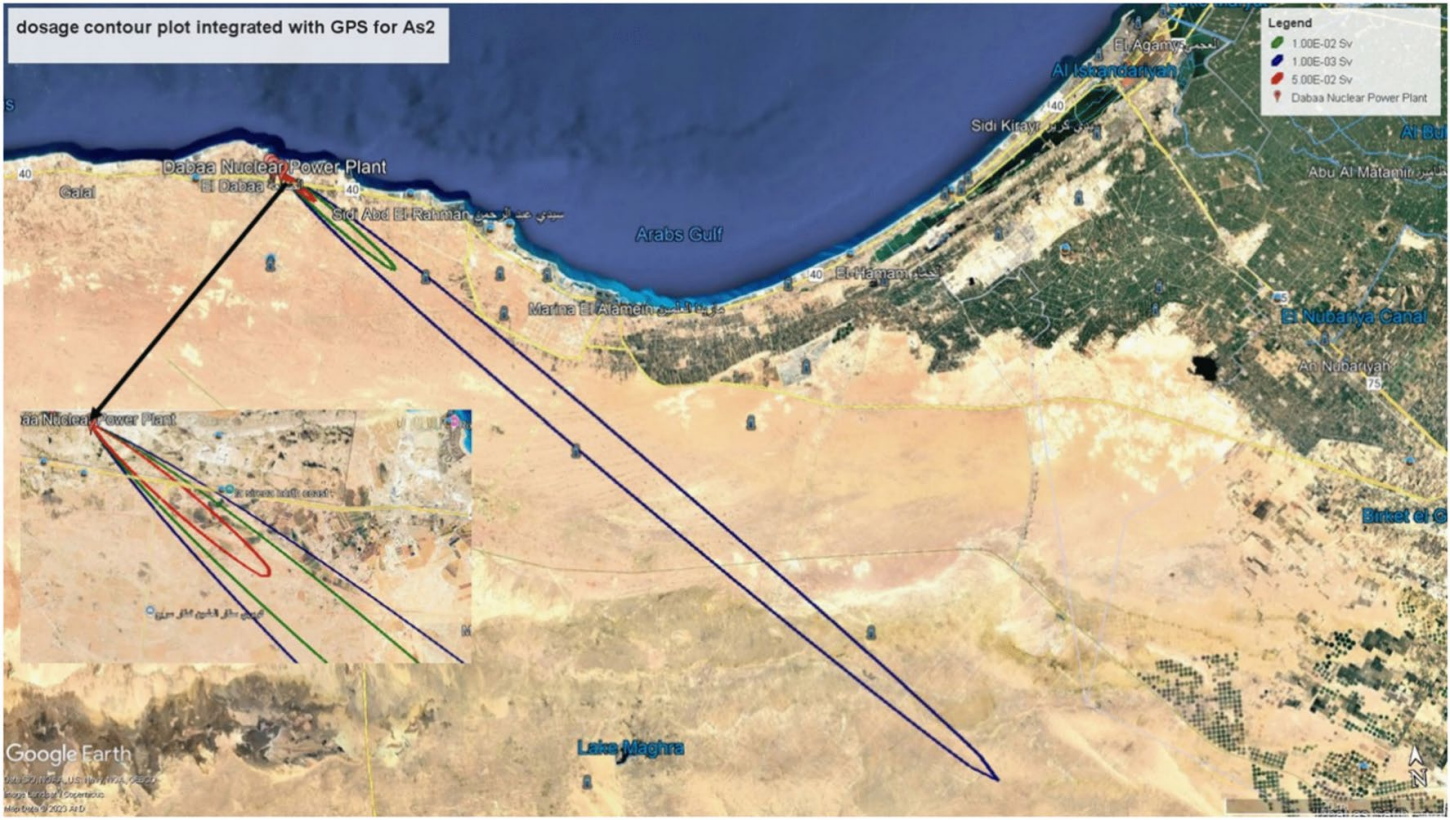
AS2 TEDE contour plots produced by integrating HotSpot^6^ with Google Earth for D stability class, wind speed of 3 m/s.

Figure 11 illustrates the contour plots produced by integrating HotSpot^6^ with Google Earth. The radiological doses of these contours are 5.00E-02 Sv, 1.00E-02 Sv, and 1.00E-03 Sv, represented by red, green, and blue outlines, respectively. The corresponding areas to these contours are 3.2 km$^2$, 27 km$^2$, and 570 km$^2$, respectively. Incident AS2 presents the most serious radiological impact, prompting Tables 6 and 7 to outline general protective and emergency response guidelines.

Table 6 details immediate response measures to be applied promptly, while Table 7 specifies intervention benchmarks for subsequent actions if initial measures from Table 6^27^. Personnel within the blue contour will be exposed to radiological doses that exceed the permissible limit of 1 mSv. Therefore, intervention measures should be implemented for individuals between the green and blue zones^28^. Personnel in the middle zone, the region between the red and green zones, are estimated to receive effective doses ranging from 10 to 50 mSv. Individuals must remain indoors to reduce exposure to surface deposits and airborne contaminants. It is essential to close all exits and openings, including windows, doors, and ventilation systems, to prevent inhalation of radioactive particles^29^. These protective measures should be implemented immediately.

Workers in the first assigned zone (red zone), with a threshold of 50 mSv, are expected to receive effective doses surpassing 50 mSv shortly after the accident. The Federal Radiation Protection Guidance for Occupational Exposures and the International Basic Safety Standards for Protection against Ionising Radiation and the Safety of Radiation Sources stipulate that workers’ radiological doses must not exceed 50 mSv in a single year and should not exceed an average of 20 mSv per year over five years. Given the circumstances above, immediate temporary relocation and evacuation of personnel in this zone should be prioritised^30,31^.

**Table 6:** Suggested actions for urgent protection.

Action	Avertable dose (generic intervention level)	Response	Case applied to the current study of AS2
Evacuation	50 mSv for no more than 1 week,	Quickly relocating people from their homes temporarily. The decision to evacuate should be based on determining the radiation dose that can be mitigated through evacuation, rather than sheltering	Any individual exists inside the red contour which extended up to 6 km corresponding to 57 mSv (see Fig. 11)
Sheltering	10 mSv for no more than 2 days	staying indoors to minimize exposure to surface deposits and airborne contaminants, and closing windows, doors, and ventilation systems to prevent inhaling radioactive particles from outdoor air	Any individual exists between the red and the green contours which extended up to 20 km corresponding to 10 mSv (see Fig. 11)
Iodine prophylaxis	100 mGy (committed absorbed dose to the thyroid)	Delivering stable, non-radioactive iodine to prevent the thyroid from absorbing radioactive iodine	Any individual exists up to approximately 40 Km in the plume direction

**Table 7:** Suggested actions for later protection.

Action	Avertable dose (generic intervention level)	Response	Case applied to the current study of AS2
Considering permanent resettlement,	1 Sv in a lifetime	Total eviction of residents with no intention of coming back for a minimum of a few years. Usually, people would be relocated into homes like the ones that were abandoned. This could entail building new infrastructure and homes	Any individual exists up to approximately 900 m in the plume direction
Initiating temporary relocation	30 mSv in a month	If the anticipated avoidable radiation dose over the next month exceeds 30 mSv, individuals should be evacuated in an organized manner for an extended but limited period (several months)	Any individual exists up to approximately 9 Km in the plume direction
Terminating temporary relocation	10 mSv in a month	Individuals will be systematically brought back once the monthly avoidable dose falls below 10 mSv	Any individual exists up to approximately 20 Km in the plume direction

An emergency plan for the current accident scenario (AS2) should include the following steps:

1. Following the accident, promptly notify key officials post-accident and alert radiological emergency teams and civil services to initiate safety protocols for each zone.
2. Setting up an off-site coordination center with appointed personnel and advisors to optimize public safety measures^29^.

Actions include deploying countermeasures, environmental surveillance, and media updates. Under the Convention on Early Notification of a Nuclear Accident, any nation experiencing an incident with potential cross-border effects must quickly alert the IAEA and concerned countries about the possible international repercussions^32^. However, in this instance, prevailing meteorological factors such as wind direction imply the risk will not extend to neighboring countries.

## Conclusion

From the modelling and simulation research conducted regarding the two theoretical nuclear incidents examined in this study, the subsequent conclusions have been drawn.

- As the distance downwind increased, both TEDE and ground deposition showed a decline. In more unstable conditions (stability class A), the highest dose peaks would be detected nearer the stack. Conversely, in stable conditions (classes E and F), higher doses tend to occur farther from the stack.
- The second incident surpassed the permissible threshold, with inhalation serving as the major contributor to TEDE.
- The CEDE analysis revealed that the thyroid is the most impacted organ by iodine accumulation.
- The influence of the ambient environment indicates that urban landscapes are likely to exhibit lower concentrations than those found in rural areas.
- Increasing the effective release altitude was found to hurt both TEDE and ground deposition. In addition, higher wind speeds effectively diminished radioactive effects. In contrast, the lower the height of the stack, the greater the TEDE and ground deposition. Moreover, precipitation, especially at higher intensities, increased both ground deposition and TEDE due to wet deposition, as raindrops capture gases and particles from the plume.

## Data Availability

All data generated or analysed during this study are included in this published article.
